# The Controversial Role of Intestinal Mast Cells in Colon Cancer

**DOI:** 10.3390/cells12030459

**Published:** 2023-01-31

**Authors:** Rosa Molfetta, Rossella Paolini

**Affiliations:** Department of Molecular Medicine, Istituto Pasteur Italia—Fondazione Cenci Bolognetti, Sapienza University of Rome, 00161 Rome, Italy

**Keywords:** colorectal cancer, mast cells, tumor microenvironment

## Abstract

Mast cells are tissue-resident sentinels involved in large number of physiological and pathological processes, such as infection and allergic response, thanks to the expression of a wide array of receptors. Mast cells are also frequently observed in a tumor microenvironment, suggesting their contribution in the transition from chronic inflammation to cancer. In particular, the link between inflammation and colorectal cancer development is becoming increasingly clear. It has long been recognized that patients with inflammatory bowel disease have an increased risk of developing colon cancer. Evidence from experimental animals also implicates the innate immune system in the development of sporadically occurring intestinal adenomas, the precursors to colorectal cancer. However, the exact role of mast cells in tumor initiation and growth remains controversial: mast cell-derived mediators can either exert pro-tumorigenic functions, causing the progression and spread of the tumor, or anti-tumorigenic functions, limiting the tumor’s growth. Here, we review the multifaceted and often contrasting findings regarding the role of the intestinal mast cells in colon cancer progression focusing on the molecular pathways mainly involved in the regulation of mast cell plasticity/functions during tumor progression.

## 1. Introduction

Colorectal cancer (CRC) is the third most common malignancy and one of the leading causes of cancer-related mortality, counting almost 1 million annual deaths world-wide, according to the International Association of Cancer Registries [1,2]. The majority of CRC cases are associated with the sporadic mutations linked to risk factors or lifestyle; 10–30% of the cases, instead, present family history, while less than 5% of patients show hereditary forms of the disease [3,4,5]. A diet rich in meat, cigarette smoke, alcohol consumption, as well as chronic inflammation in patients with inflammatory bowel disease (IBD) all represent critical independent risk factors for CRC development [6,7,8,9,10]. CRC pathogenesis and progression are driven by distinct genetic features and events of genomic instability which lead to different CRC phenotypes characterized by chromosomal instability, the hypermethylation of promoter CpG island sites (CpG island methylator phenotype, CIMP), and the high level of microsatellite instability [3,11,12]. Along with CRC classifications based on the features of cancer cells, including microsatellite instability and TNM (Tumor–Node–Metastasis) staging, the WHO has introduced the “immunoscore” as a prognostic value for predicting disease-specific recurrence and survival [13,14,15,16]. Among the parameters, this score includes the density of tumor-infiltrating cytotoxic and memory T cells, which are associated with favorable prognosis [17], whereas scores reporting the frequency of innate immune cells, including mast cells (MCs), are limited [18]. 

Initially neglected, MCs are progressively becoming crucial players in CRC because increasing evidence supports their capability to affect tumor progression [19,20,21]. 

Nonetheless, despite the many works that have been published in the last year, the ultimate role of MCs in tumors is far from being understood. The findings are characterized by apparently contradictory data, which actually are consequent of the plastic nature of MCs that are extremely sensitive to microenvironmental cues to which they suddenly respond. Hence, the effect of MCs cannot be limited to the dichotomy presence/absence but it is caused by their localization, density, activation and degranulation state, secretion of cytokines and/or proteases, and proximity to other immune and cancer cells [22]. MCs could also represent an important tool to predict cancer outcome, but, before they can be employed as prognostic/predictive markers or even as targets for novel therapeutic approaches, a deeper characterization of their biology and the identification of the specific profiles associated with their activation and localization are necessary.

In this review, we provide an overview of the features of intestinal MCs and their potential roles in the development of CRC with a particular focus on the interplay between MCs and tumor cells in tumorigenesis.

## 2. Phenotypic and Functional Heterogeneity of Intestinal MCs

MCs are innate immune cells that originate from bone marrow precursors, enter the circulation as committed progenitors (MCp), and migrate into peripheral tissues where they further differentiate under the influence of the local microenvironment [23]. Mature MCs are strategically distributed in close proximity to blood and lymphatic vessels as well as nerves to respond to pathogens and other ingested or inhaled agents [24,25,26]. Indeed, MCs express a wide array of receptors that allow them to recognize invading pathogens and respond to different stimuli coming from the microenvironment [27,28]. MCs are able to integrate environmental signals and in response to all of them release different granule-stored or newly synthetized chemical mediators and cytokines [29,30]. 

In the gut, MCs are the main sentinels of the host defense involved in the maintenance of homeostasis as well as in orchestrating local inflammation [31]. 

Compared to other peripheral tissues, the gut is a peculiar site since it presents an abundant reservoir of murine MCp that constitutively home to this organ thanks to the expression of the integrin α4β7 and the chemokine receptor CXCR2 [32]. Committed murine MCp in the gut have been characterized as Lin^−^c-Kit^lo^FcεRIα^lo^α4β7^+^ cells and under the influence of local factors, differentiate into fully mature MCs upregulating FcεRIα while downregulating α4β7 [33].

**Figure 1:**
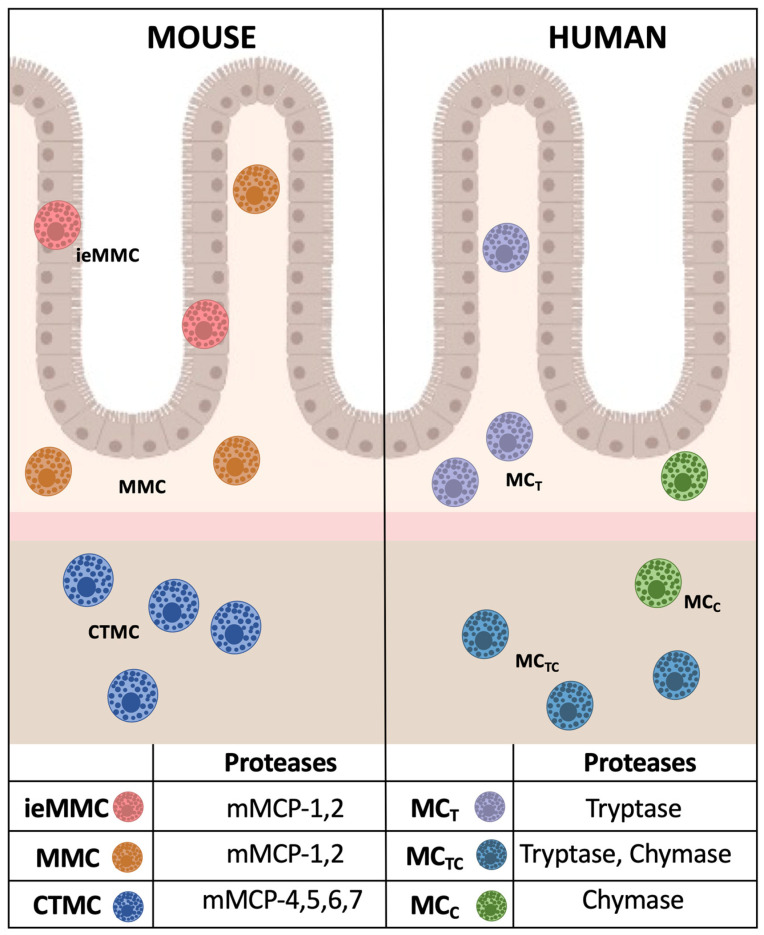
Mast cell subsets in murine and human gut. Mast cell subsets identified in murine intestine (**left**) and human intestine (**right**), and their distribution are represented with different colors. Proteases expressed by the different MC subsets are listed in the table below. ieMMC: intraepithelial mucosal mast cells, MMC: mucosal mast cells, CTMC: connective tissue mast cells, MC_T_, MC_TC_, MC_C_: Mast cells expressing tryptase, tryptase and chymase, chymase only, respectively.

The two main subsets of MCs have been identified in the murine intestine based on the expression of MC proteases [34]. Mucosal MCs (MMCs), positive for MCP-1 and MCP-2, are found in the lamina propria close to the epithelium and produce lower levels of histamine and higher amounts of cysteinyl leukotrienes compared to the larger connective tissue MCs (CTMCs) found in the submucosa (Figure 1, left panel). CTMCs contain the chymase mMCP-4, the elastase mMCP-5, the tryptases mMCP-6 and -7, and the metallopeptidase carboxypeptidase A3 (CPA3) in their granules [34,35]. A third MC subtype was recently identified in mice: the interepithelial mucosal MCs (ieMMC) [36]. Although much remains to be learned about the differing functions of lamina propria and ieMMCs, both of them are rare in normal mouse intestinal mucosa but are increased during the immune responses to intestinal helminth infections and in food allergies [36]. 

MC heterogeneity was also reported in the human gastrointestinal tract (Figure 1, right panel). The tryptase-positive and chymase-negative MC population is mainly present in the lamina propria (MC_T_), while the main MC subset in the submucosa (MC_TC_) is characterized by the expression of both enzymes [34,37,38]. A rare population of MCs exclusively expressing chymase was also identified in both lamina propria and submucosa [38], but its role is still unclear. 

However, mouse and human MC classifications are simplistic since they do not reflect the high level of intestinal MC plasticity due to the constant change in the local microenvironment. Using singe-cell RNAseq technology, Dwyer and coauthors have elucidated tissue-specific MC peculiarity, revealing at least three distinct connective tissue MC subsets [39].

## 3. Role of Intestinal MCs in Immune Homeostasis and Infections

Different MC subsets may regulate the intestinal barrier function in homeostatic conditions and upon infections. Mice deficient for MCP-4 show a reduced intestinal permeability and epithelial cell migration [40], highlighting a role for CTMCs in homeostatic conditions. 

Upon parasite infection, a high degree of MC plasticity is well underlined. MMC is the main subset that expands and increases the intestinal permeability to facilitate the expulsion of nematode through the action of MCP-1 [41]. During the acute phase of the *Trichinella spiralis* infection, MCs dramatically increase in number and change from a connective to a mucosal phenotype, mainly expressing the chymase family members MCP-1, MCP-2, and MCP-10. MCP-1 directly participates in the clearance of infection since the delayed expulsion of the adult helminth and increased deposition of muscle larvae have been reported in MCP-1-deficient mice [42]. In the recovery phase, MCs slowly revert back to their initial protease phenotype and restore their physiologic number [43]. 

By contrast, Shin and co-authors underly a selective role of connective tissue MCs in the chronic phase of *T. spiralis* infection. Indeed, they showed that the tryptase MCP-6 is important for the recruitment of eosinophil to *T. spiralis* larvae and for the infection’s clearance [44]. 

A different scenario characterizes an acute infection with a high-dose of the nematode *Trichuris muris* that results in a persistent accumulation of MCs with mucosal phenotype in the large-intestinal epithelium [45], having long-term consequences on the barrier integrity.

The apparent discrepancy between the two infection models can be explained by the different location of an infection (small versus large intestine) and/or by a selective change in the resident microbial communities. 

The MC plasticity, intended as the ability to rapidly sense a changing environment and consequently adapt to the specific received triggers, can explain the activated MC phenotype described in different human gastrointestinal disorders. Several studies have reported an increased number of MCs in the mucosa of patients affected by celiac disease, IBD, irritable bowel syndrome, and mucosal MCs resulted to be fundamental mediators of the pathology-associated inflammation [46]. 

Of note, the large majority of intestinal MCs are in direct contact with nerves and the MC/nerve crosstalk provides a neuroimmune network necessary to control the physiological and pathological response of the gut [47,48]. 

MCs locally release histamine and tryptase that may impact on the intestinal neurons through histamine receptors and the proteinase-activated receptor (PAR-2), thus inducing the muscle contraction and pain that characterize intestinal inflammation [49,50]. On the other hand, upon cytokine priming, human MCs express neurokinin receptor 1, thus becoming responsive to substance P, while murine MCs can sense several neuropeptides including adenosine triphosphate, somatostatin, calcitonin gene-related peptide, and substance P [51].

Even though a direct effect of neuropeptides on MC activation is more evident in murine models, the increased neuropeptide amounts in IBD patients suggests a possible role for MC/intestinal neuron cooperation in the pathogenesis of the disease.

## 4. The Ambiguous Role of Intestinal MCs in Colorectal Cancer Development

The link between the persistent inflammation and tissue transformation is now solid and best represented by CRC development in patients affected by IBD [50]. Indeed, the presence of morphologically activated or degranulated MCs in the colon during the florid phase of the inflammatory process suggests their possible role in the transition from inflammation to carcinoma.

However, the precise role of the different intestinal MC subsets during CRC development is still a matter of debate [19]. 

### 4.1. Evaluating MC Infiltration in Human Patients with CRC

By in situ detection of MCs, several studies have highlighted a correlation between an increased MC density and a poor prognosis [52,53,54,55,56]. In a more recent finding, a link between the frequency of circulating MC progenitors and advanced stages of CRC disease was also reported [57]. 

Of note, MCs have been observed mainly at the tumor margins and at peri-vascular regions [54,55], and their presence was associated with an increased blood vessel density in the tumor microenvironment (TME) [58].

Despite the finding that support a pro-tumorigenic role for MCs, other studies have reported a correlation between a high MC density and better clinical outcomes [59,60,61]. 

It is likely that age, sex, and racial disparities [62] as well as the method(s) used for detecting the MC frequency in tissues may account for the described discrepant results.

Regarding the phenotypic features of MCs among normal tissues and colorectal cancer, Tan and co-authors have found no differences in the proportion of the MC_TC_ subtype and of the MC_T_ subset, making their percentage approximately 75% and 25%, respectively [60]. 

However, most studies on the density of tumor-infiltrating MCs were performed through in situ detection of tryptase-positive cells in the invasive front of colonic adenocarcinomas without discrimination between MC_TC_ and MC_T_ subsets [55,58,63,64]. Tryptase represents one of the most powerful angiogenic mediators released by human MCs [65]. In a human colon carcinoma cell line, Yoshii et al. demonstrated that tryptase activates PAR-2 on tumor cells and that this activation in turn led to the production of PGE2 and the induction of cell proliferation [66]. 

However, no conclusive data exist about the role of human tryptase in the carcinogenesis process, emphasizing the need to examine the impact of MCs and their proteases in CRC development from different perspectives.

### 4.2. Mouse Models to Study the Role of MCs in CRC Development

**Figure 2:**
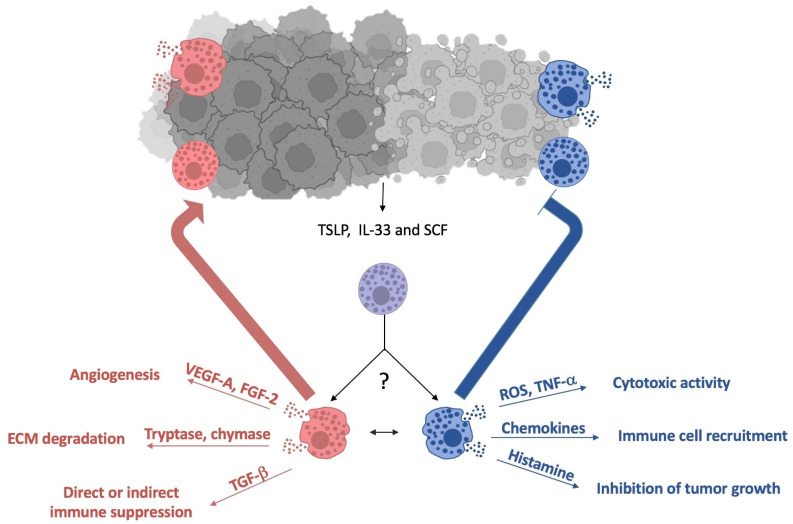
The dichotomous role of MCs during CRC. MCs infiltrating colonic tumor can directly interact with cancer cells or recognize tumor-released factors, such TSLP, IL-33 and SCF, tuning their phenotype/activity. Different MC subsets can exert either pro-tumorigenic (red arrow) or anti-tumorigenic (blue arrow) properties through the secretion of preformed and newly synthesized mediators, the main of which are depicted. TSLP: thymic stromal lymphopoietin; IL-33: Interleukin-33, SCF: stem cell factor, VEGF: vascular endothelial growth factor; FGF: fibroblast growth factor, TGF: transforming growth factor, ROS: reactive oxygen species, TNF: tumor necrosis factor.

Several mouse models have been used to dissect the role of intestinal murine MCs during colon inflammation and tumor progression. Depending on the specific tumor model and on the experimental settings under investigation, heterogeneous MC functions have been proposed either to sustain or resolve the tumor progression (Figure 2) [19,67]. 

Among the studies supporting a role for MCs in the development of CRC, Gounaris and co-authors demonstrated that MCs and their progenitors accumulate in the colonic polyps of adenomatous polyposis coli mutant mice (APC^Δ468^) in a T cell-independent manner [68]. They observed that the depletion of MCs, by using APC^Δ468^ mice lethally irradiated and reconstituted with c-kit defective BM (Kit^W-sh/Wsh^), leads to a decrease in the frequency and size of colonic tumors, suggesting that MCs and their soluble mediators are the essential components for polyp development. In particular, the authors proposed that the MC-derived tumor necrosis factor α (TNFα) acts in an autocrine fashion to amplify the local MC pool at the site of the tumor’s formation and directly contributes to the adenomatous polyp growth [68]. 

These results are consistent with a previous study demonstrating a reduced susceptibility of MC-deficient B6Kit(W)/Kit(W-v) mice to chemically induced intestinal tumors [69], and with other evidence suggesting that MCs acquire a pro-tumorigenic role during the development of colitis-associated CRC [70,71]. 

However, there is also evidence in which MCs appear to play a protective role in CRC tumorigenesis. For example, Sinnamon and co-authors demonstrated that in the absence of MCs, the frequency and size of adenomas increased in mice carrying a heterozygous mutation in the adenomatous polyposis coli gene (ApcMin/+ mice) [72]. Similarly, Haribabu and colleagues reported that an accumulation of MC in the small intestine of ApcMin/+ mice mediated immune surveillance through a selective recruitment of anti-tumor CD8+ T-cells, reducing the intestinal tumor burden [73]. 

A more recent work from Sakita and colleagues provided evidence that in vivo MCs could either promote or inhibit the development of colon tumors according to the type of microenvironment stimuli, being tumorigenic in colitis-induced CRC and protective in murine models of sporadic CRC [74]. 

Finally, it is important to underline that murine small intestinal tumors have a different immunological milieu than colonic tumors. In the case of MC subsets, during the progression from adenoma to carcinoma, a decreased number of ieMMCs have been reported in colorectal lesions but not in small intestinal tumors [75].

Thus, it is likely to be concluded that the MC activity during the CRC development may impact either in a beneficial or harmful fashion depending on the genetic background, specific tumor models, and regional diversity in the microenvironment composition. 

## 5. Gut Microenvironment “Education” and MC Switch during Inflammation and Colon Cancer Development

Similar to the case of macrophages, MCs undergo to a sort of “microenvironment education” under the influence of cytokines, growth factors, and microbial components that tune the MC phenotype and functions in homeostatic and inflammatory conditions [22,76]; however, the molecular mechanism(s) are still undefined.

On the other hand, once activated, the MCs recruited in the initial phase of tumorigenesis release several mediators that directly modify the TME and/or indirectly regulate the local immune response [20,77]. 

### 5.1. Crosstalk between MCs and Microbiome

Emerging evidence support a mutual influence between MCs and gut microbiota, as is well documented by the use of germ-free (GF) mouse models [76,78,79]. Commensal bacteria through the interaction with Toll-like receptor (TLR) 2 and 4 promote the expression of CXCR2 ligands by intestinal epithelial cells, which, in turn, is responsible for MC maturation and their recruitment into intestine [78]. Accordingly, upon oral sensitization with ovalbumin GF mice did not develop the symptoms of a food allergy despite the production of high IgE levels [79]. 

The ability of bacteria and fungi to elicit an MC activation is further supported by the in vitro data demonstrating that a coculture of MCs with some bacteria strains or Candida Albicans induce MC degranulation and pro-inflammatory cytokine production, as well as the release of VEGF [76]. MC activation, in turn, may facilitate the elimination of microorganisms either through the secretion of pro-inflammatory cytokines or the production of MC extracellular traps [80,81].

On the other hand, other microorganisms are able to inhibit FcεRI-mediated intracellular signals in vitro and in vivo, thus dampening the MC functions [76]. 

The ability of gut microbiota to shape the MC functions suggests that dysbiosis may cause the intestinal accumulation of MCs in inflammatory conditions, including celiac disease, Crohn’s disease, and ulcerative colitis, and that the increased intestinal permeability may further facilitate an MC-microbiota crosstalk [50]. 

Experimental evidence from the past years have also highlighted a key role for the intestinal microbiota in malignant gastrointestinal diseases [82]. Remarkably, similar alterations in the microbiota composition (e.g., higher abundance of *E. coli*) has been reported in Crohn’s disease individuals and in patients affected by colon cancer [83,84], suggesting that a shared dysbiosis may contribute to IBD-associated CRC. 

However, the microbial mechanisms associated with human CRC pathogenesis during the crosstalk between MCs and microbiome remain undescribed. 

### 5.2. Bidirectional Interaction between MCs and Cancer Cells

During an inflammatory state, a molecular mechanism behind the phenotypic/functional switch of MCs could be the result of their interactions with epithelial cells and/or the soluble factors that these cells produce, including the alarmins thymic stromal lymphopoietin (TSLP) and interleukin (IL)-33 that deserve a particular mention [85,86,87,88]. 

In the intestine, TSLP and IL-33 control the balance between the host defense and wound repair [89,90]. In particular, the capability of murine MCs to resolve IL-33-mediated inflammation appears to be critical in promoting tissue repair through MC-mediated protease and cytokine production [70,90]. 

TSLP and IL-33 released either by gut epithelial or immune cells can also play a direct role in tumorigenesis [91,92]. Using a xenograft mouse model of colon cancer, Yue and coauthors demonstrated that the peritumoral administration of TSLP reduces tumor growth by inducing the apoptosis of human colon cancer cells in a TSLPR-dependent manner [91]. 

On the other hand, during CRC progression, the abnormal expression of IL-33 in the TME activates tumor stroma to promote intestinal polyposis [92]. 

Transformed cells by releasing IL-33 can then elicit pro-inflammatory functions in MCs to reprogram them into a pathogenic state [93]. 

In the context of small bowel cancer, during the transition from inflammation to cancer, it was recently reported that distinct MC subsets expand and guide tumor progression [94]. Specifically, Saadalla et al. reported that mucosal MCs, the same MC subtype that expand during an acute *Trichinella spiralis* infection, accumulate in benign polyps in the presence of IL-10, IL-13, and IL-33 and are maintained in an IL-10-dependent manner. However, during the transition of polyps to adenocarcinoma, a different subset characteristic of connective tissue MC expands and accumulates inside the tumor stroma in an IL-33-dependent manner [94], supporting cancer’s ability to change the MC activity toward a pro-cancer function. Accordingly, in the ApcMin/+ mouse model, an IL-33 deficiency inhibited the intestinal tumor burden and decreased the MC density, as well as the release of MC-derived proteases and cytokines [92,95]. 

Moreover, in an azoxymethane (AOM)-induced colonic tumor model, the MCs recruited to epithelial cells during an acute inflammation play a role in the resolution of colon damage but acquire a pro-tumorigenic role during epithelial cell transformation [70]. 

It is likely to be concluded that MCs acquire a different behavior when faced with normal, damaged, or transformed epithelial cells. Such deviated immune responses would benefit the progression of cancer through promoting chronic inflammation molecular pathways over cytotoxic pathways. 

MCs represent an abundant source of several angiogenic and lymphangiogenic factors that have been shown to play a role in inflammatory and neoplastic angiogenesis [96,97,98,99]. Of note, the important role of MCs as producers of the angiogenic factor VEGF-A has been recently underlined by a transcriptomic analysis of tumor-infiltrating myeloid cells in different human cancers, including CRC [100].

MC-secreted factors facilitate tumor vascularization not only by a direct angiogenic effect but also by stimulating MCs themselves and other inflammatory cells in the TME to release novel angiogenic mediators and cytokines. 

MCs release matrix metalloproteinases 9 and specific proteases (tryptase and chymase) that degrade the components of the extracellular matrix, promoting the spread of a tumor and metastasis [98]. Moreover, by releasing adenosine and amphiregulin, MCs can suppress the protective immune responses against cancer [101,102]. 

On the other hand, MCs represent the only cell type able to store preformed TNF-α in their granules [103] and, upon degranulation, can also exhibit anti-tumor activity through direct tumor cell cytotoxicity mediated by TNF-α and reactive oxygen species. 

To deeply investigate the interplay between colon cancer cells and MCs, by using 2D and 3D human coculture models, Yu and co-authors provided evidence that support a bidirectional crosstalk in which cancer cells, mainly producing stem cell factor (SCF), support MC recruitment, while MCs release pro-tumorigenic mediators and increase colon cancer growth [104]. They also compared the transcriptomic profile of colon cancer-cocultured human MCs versus control MCs [105]. A list of deregulated genes has been identified, including *MMP-2*, *VEGF-A*, *PDGF-A*, *COX2*, *NOTCH1*, and *ISG15*, which all contribute to the enrichment of cancer-related pathways. To better validate the complex interaction between MC and TME, a 3D multicomponent coculture should be developed by the incorporation of other stromal/immune cells. 

By a similar approach, utilizing whole-genome gene expression data from both mouse models and human cancer patients, Ko and coauthors demonstrated that the expression profile of “MC-dependent genes” (deregulated by MC deficiency but largely recovered upon MC engraftment) differs between normal and tumor from lung, breast, and colon tissues [106]. 

However, further studies are needed to validate the exact role of individual MC-dependent genes in CRC development.

### 5.3. Crosstalk between MCs and Other Immune Cells during CRC Progression

The activation of MCs leads to the release of a plethora of factors that act on other immune cell types in the TME and influence their recruitment, rate of proliferation, and their state of activation, differentiation, and polarization [20].

Eissmann and co-authors recently demonstrated a vicious signaling axis between IL-33, MCs, and macrophages to sustain angiogenesis and the growth of gastric cancer: tumor epithelial-derived IL-33 activates MCs to produce a chemotactic cytokine expression signature, which promotes a selective accumulation of tumor-associated macrophages (TAMs) [107]. They found that the ablation of MCs or macrophages in tumor-bearing mice were associated with a vascular collapse and tumor hypoxia. Importantly, in gastric cancer patients, this MC activation signature correlates with decreased patient survival. 

In CRC, earlier studies have demonstrated the presence of both MC and TAMs in the stromal tumor front [63,108]. They are located in close proximity and promote tumor growth-releasing pro-inflammatory and angiogenetic growth factors [108,109].

Accordingly, using the piroxicam/IL-10−/− mouse model of progressive colitis, Khan and co-authors found that MCs and TAMs may communicate and through their crosstalk, promote cancer cell invasion [110]. Upon the activation of the PI3K/AKT signaling, cascade MCs acquire the ability to attract CD11b+ cells that in turn promote tumor cell proliferation and invasion. 

Taken together, these findings support the hypothesis that the progression from chronic inflammation to CRC can be influenced by the interaction between stromal, MCs, and TAMs and that the inhibition of PI3 Kinase could antagonize such a vicious loop.

In murine polyposis and human CRC, MCs also established with Treg an intricate interaction that regulates the functions of both cell types in a reciprocal manner [111,112]. Interestingly, MCs induce conventional Tregs to switch function, generating a potent immune suppressive but proinflammatory Treg population that shuts down IL-10 and promotes MC expansion and degranulation [113]. 

Another mechanism by which MCs could promote immune evasion is through the interaction with myeloid-derived suppressor cells (MDSCs). 

Activated MCs, by the release of nitric oxide, IL-6, and TGF-β, increases the recruitment and activity of MDSC, which in turn results in a strong inhibition of T-cell proliferation and consequent tumor-induced tolerance [114]. More specifically, in the APCΔ468 murine model, it was reported that MCs support the MDSC activity in driving the immune escape with a subsequent increase in the polyp development [115,116].

Since MDSCs can enhance MC activation [116], within the TME, a pro-tumor positive feedback loop between MC and MDSC can be also envisaged and likely contributes to immune evasion. 

## 6. Concluding Remarks

Many questions related to the role of intestinal MCs in CRC progression remain unresolved. 

To investigate the role of MCs within the immunologic milieu of CRC, “mast cell-deficient” mice have been derived primarily on the C57BL/6 background and, to a lesser extent, on BALB/c, and work with such animals produces results that might be mainly restricted to those strains of inbred mice. Additionally, most findings came from murine models of c-Kit mutations that do not affect exclusively MCs but can also influence the basophil numbers and functions, as well as intestinal γ/δ T cells. 

It is also important to mention that a non-negligible proportion of genetically engineered mouse models in CRC research (e.g., Apc-mutated) develop tumors predominantly in the small intestine, which is in sharp contrast to the human situation. Moreover, murine small intestinal tumors have a different immunological milieu than colonic tumors and murine MCs might therefore affect the adenoma-carcinoma progression differently depending on the intestinal region considered.

Thus, advanced functional studies in MC-deficient mouse models and the second generation of genetically engineered mouse models for intestinal cancer (e.g., tumor initiation plus Cre-mediated immune cell manipulation) will be required for ultimately determining the MC role in intestinal cancers. In particular, considering the great relevance of MC heterogeneity in intestinal cancer, it will be important to choose mouse models in which the specific targeting of all, or of one specific, MC subtypes is assured.

Another current goal is to better understand whether progenitors generate distinct intestinal MC subsets, meaning anti-tumorigenic and pro-tumorigenic MCs, and/or whether individual MCs have sufficient plasticity to exhibit distinct features based on their responsiveness to particular TME signals. The application of single-cell RNA sequencing platforms will allow for a high-resolution characterization of an intestinal MC signature that accompanies a colonic tumors’ initiation and growth and may help to solve these questions.

Mast cell secretory granules contain various bioactive mediators, including tryptase, chymase, and carboxypeptidase A. However, the contribution of specific MC proteases during CRC progression is still largely unclear. 

Further studies will be also needed to fully investigate the effects of the microbiota on intestinal MC functions in CRC tumorigenesis.

Finally, a better characterization of intestinal MCs at various stages of colonic disease would help to define to what extent one can safely enhance the positive functions of MCs, or inhibit their harmful activities, in order to offer novel potential targets for a therapeutic intervention in CRC progression.

## Abbreviations

Adenomatous polyposis coli (APC); azoxymethane (AOM); colorectal cancer (CRC); inflammatory bowel disease (IBD); mast cell (MC); interleukin-33 (IL-33); mast cell progenitors (MCp); proteinase-activated receptor (PAR-2); stem cell factor (SCF); tumor-associated macrophage (TAM); tumor microenvironment (TME); tumor necrosis factor (TNF); thymic stromal lymphopoietin (TSLP).
